# GIS-based landform classification of Bronze Age archaeological sites on Crete Island

**DOI:** 10.1371/journal.pone.0170727

**Published:** 2017-02-21

**Authors:** Athanasios V. Argyriou, Richard M. Teeuw, Apostolos Sarris

**Affiliations:** 1 School of Earth and Environmental Sciences, Centre for Applied Geosciences, University of Portsmouth, Portsmouth, PO1 3QL, United Kingdom; 2 Laboratory of Geophysical—Satellite Remote Sensing & Archaeo-environment, Institute for Mediterranean Studies (I.M.S.) / Foundation of Research & Technology (F.O.R.T.H.), Melissinou & Nikiforou Foka 130, Rethymno, Greece; New York State Museum, UNITED STATES

## Abstract

Various physical attributes of the Earth’s surface are factors that influence local topography and indirectly influence human behaviour in terms of habitation locations. The determination of geomorphological setting plays an important role in archaeological landscape research. Several landform types can be distinguished by characteristic geomorphic attributes that portray the landscape surrounding a settlement and influence its ability to sustain a population. Geomorphometric landform information, derived from digital elevation models (DEMs), such as the ASTER Global DEM, can provide useful insights into the processes shaping landscapes. This work examines the influence of landform classification on the settlement locations of Bronze Age (Minoan) Crete, focusing on the districts of Phaistos, Kavousi and Vrokastro. The landform classification was based on the topographic position index (*TPI*) and deviation from mean elevation (*DEV*) analysis to highlight slope steepness of various landform classes, characterizing the surrounding landscape environment of the settlements locations. The outcomes indicate no interrelationship between the settlement locations and topography during the Early Minoan period, but a significant interrelationship exists during the later Minoan periods with the presence of more organised societies. The landform classification can provide insights into factors favouring human habitation and can contribute to archaeological predictive modelling.

## Introduction

During the Middle Minoan period of the Bronze Age period in Crete, there appears to have been a widespread increase of sites in low elevation areas suitable for arable farming. Systematic archaeological surveys across eastern and central Crete have examined the settlement dynamics and in all cases highlight a generalised population movement from high elevation areas with limited arable land to lower elevation areas, particularly plains favourable to cultivation and efficiency in irrigation [1]. Most of the studies hypothesized that this population movement tendency was caused by economic and political reasons but only a few of them considered the possibility that this tendency could be a result of environmental conditions and resource exploitation [2,3,4]. Mountainous sites at higher elevations (~600–800 m) seem not to attract further exploitation during the Middle Minoan (and were rarely re-occupied permanently during the later Minoan periods). That was perhaps due to the development of new practices for intensive agriculture which could not be applied in steeply sloping terrain [1]. As a result, people relocated to lower elevations (~300 m) and plains, which have high agricultural and irrigation potential to support population growth, as in Kavousi and Vrokastro districts [5,6].

This study aims to examine the hypothesis outlined above by studying settlement dynamics and landform characteristics during the Minoan periods, to check whether the population movement to lower elevation areas was a random tendency, or was interlinked with agricultural practices. It also examines the more general trend that the clusters of settlements followed, despite their local “micro-regional identity” [5]. Investigation of heterogeneity in geological and geomorphological properties can lead to the quantification of landscapes and to a better understanding of their complexity [7,8,9].

The Earth’s surface consist of large geomorphic features (e.g. plains, mountain ranges), through to smaller features (e.g. valleys) and their component landforms, such as valley slopes, floodplains and terraces [10]. Such landscape classification information can be of use for archaeological studies because the two sets of data can be interlinked to gain insights into the factors driving settlement evolution [11,12]. Topographic prominence was one of the first approaches to find application on archaeological studies while examination of other geomorphometric parameters followed (e.g. terrain ruggedness, amplitude of relief). Such was the case with archaeological studies of Cyprus, where an evaluation of the exposure of cultural heritage sites to natural hazards took place [13,14,15,16,17]. Most of these studies were mainly investigating the local or relative topographic position of archaeological sites in the landscape [18,19].

More recently, the development of computer technology, software packages and free access to datasets attracted the interest of analysts to develop computer algorithms to examine geomorphometric attributes and the topography of the Earth’s surface [20,21,22]. In particular, in recent years there has been an increase of interest in use of GIS-based analyses to classify landforms, over various scientific fields such as geomorphology, geology, agriculture [23,21]. Despite that, only a few studies exist with applications of archaeological interest [24,19]. The majority the studies consist of automated or semi-automated approaches, mostly evaluating homogeneous landscapes [25,26]. In contrast, this study examines both homogenous and heterogeneous landscapes. The information derived by terrain analysis, via DEMs and geomorphometrics, offers to analysts a powerful approach to describe the topographic position of features [27,21].

Understanding the relationship between the types of archaeological sites (e.g. settlements, defending sites or burial sites) and their surrounding landscape type is an important aspect of archaeological investigations. Landscape geomorphometrics can reveal insights into variations in the distribution of settlements over time [28,12]. Archaeological landscapes can be quantified by using the topographic position index (*TPI*) (or difference from mean elevation, *DIFF*), which can classify the landscape, both in terms of slope position and landform categories, into morphological classes based on the geomorphology [29,30,31]. In addition, the deviation from mean elevation (*DEV*) can be useful tool for geo-archaeological studies, because it highlights the subtle topographic features [32]. These two approaches are investigated in this study because they can be significantly enlightening from an archaeological perspective. The periods of the Early Minoan (5,600–4,000 BP), Middle Minoan (4,000–3,600 BP) and Late Minoan (3,600–3,000 BP) are the focus of this study [6,33,34].

## Study area

**Fig 1:**
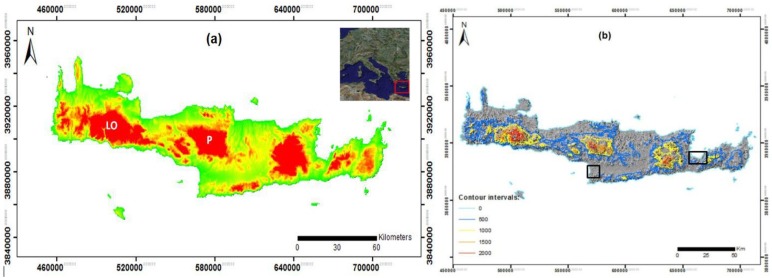
(a): The island of Crete, with red tones highlighting mountainous relief (LO: Lefka Ori; P: Psiloritis); (b) Crete shaded relief and contour intervals. The black boxes indicate the case study sites.

Crete is located in the southern part of Greece and has a significant archaeological heritage. Evidence from stone tools reveals human presence on the island of Crete as early as 130,000 years ago. However, evidence for modern human presence dates to 10,000–12,000 years ago and it was not until the Neolithic period (8500–4900 BP) when the first signs of advanced agriculture appeared in the Aegean, to open the way for the subsequent emergence of the Minoan civilization (5,600 to 3,000 BP), which is considered as the birthplace of the earliest “high culture” in Europe [35,36,37] (Fig 1A). The Phaistos, Kavousi and Vrokastro districts were selected as case study sites because of their rich archaeological heritage (Fig 1B). The information derived from past archaeological surveys is sufficiently detailed for the analysis of variations in settlement locations during the Minoan periods [6,33,38]. Earlier prehistoric geomorphological conditions were not considered in this methodological framework, as a freely-available Digital Elevation Model (DEM) of recent years is being used in the analysis: further research is needed to determine palaeo-geomorphological features of the study region. In general, the landscape of the study region has remained stable since the earliest phases of human settlement, with the main terrain features being formed in the Pleistocene (i.e., mostly within the past 2 million years) [39]. Regarding palaeo-climatic conditions, at present no conclusive observations exist for Crete from Bronze Age to the present. The role of climatic fluctuations during that period is in general an unexplored field, although a few studies have attempted to evaluate climatic changes with societal developments in areas around eastern Mediterranean [40,41]. [34] reviewed existing indirect measurements of palaeo-climate, so-called climate proxies (e.g. stable isotopes, fossil microshells), with few climatic fluctuations (drought or wetter conditions) being observed in Crete during the Minoan period. As [34] describes, the existing data is unevenly distributed, with some periods having one or two proxies and others by seven or eight. That highlights the importance of integrating climate and environmental history because a relationship between minor Alpine glacial advances in Europe and periods of extreme weather in the Aegean seems to have existed in the study region [39].

## Minoan land use patterns

During Early Minoan, in the case of the Kavousi-Vrokastro district, the settlements are preferably established on heterogeneous landscapes and many are found at higher elevations with complex rough terrain, mainly hills and ridges [6]. The Mirabello Bay area consists of small coastal valleys: most of the settlements seem to be placed on slopes rather than on the best (flat, lowland) agricultural land [42]. The site pattern was defensive, providing lines of sight between the settlements and out across the Mirabello gulf. Thus the Vrokastro site pattern may be in some accord with [43] suggestion that “people were forced to look for their safety in barely accessible places”. During this period it seems there was a dichotomy between occupying low-lying coastal sites and upland locations, indicating a tendency of the population to occupy diverse types of terrain, seeking to gain control of water sources and sites with the greatest visibility [6]. The evidence for settlement hierarchy during this period has been doubted [44], with clusters of settlements found around the arable coastal basins and not around large settlements.

The Middle Minoan is characterized by settlements in homogeneous landscapes, such as the Kavousi plains, which were well-suited for cultivation [38]. There appears to have been a movement of population into the lowlands and arable upland areas close to water resources, with an associated increase in number of settlements near the best agricultural land during Middle and Late Minoan [6,33,38]. [5] notes that the pattern of settlements exploitation in the Kavousi area is in accordance with the Argolid model during this period, with concentrated population around arable areas, similar to the expansion pattern of small farms inland in Chania district.

Between the Early and Middle Minoan, population growth occurred in the Gournia valley, with continuous expansion of settlements and coastal trading interests [6]. In addition, during the Middle Minoan there is exploitation of the landscape at relatively high elevations, between 450 m and 700 m above sea level. River terraces and bedrock of conglomerates and marl provided fertile soil for cultivation of the Gournia valley, along with fault fractures that provided water access via springs at higher elevation [6].

In the Late Minoan an overall reduction of the number of settlements is observed, especially in upland sites. Despite that, rural settlement is oriented to arable land and water resources, preferentially between 50–200 m elevation range, while a retreat from coastal zones (0–50m) seems to have occurred [6]. An event that might be able to explain this reduction of settlements is the impact of the Theran eruption, with evidence of a thick layer of tephra/ash found in the Mochlos area, near Gournia. Population centralization is also observed during the Late Minoan, for example at Pyrgos or Gournia. Some new large building structures during this period can be found (e.g. around the Istron river valley) which appear to be linked to trade routes, access to arable land and water resources [45].

## Materials and methods

### *TPI* and *DEV* analyses for various neighbourhood sizes

**Fig 2:**
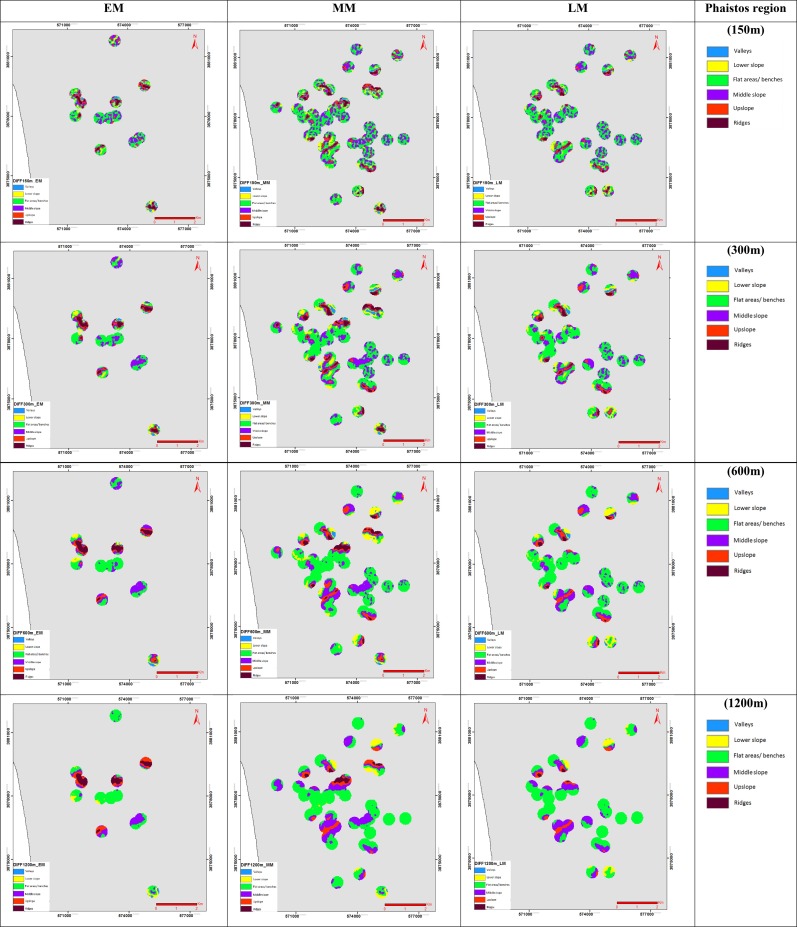
*TPI or DIFF* for EM, LM and MM period on Phaistos region, with six morphologic classes for the neighbourhood sizes: a) 150 m; b) 300 m; c) 600 m; d) 1200 m (see S1 Fig for Kavousi-Vrokastro region).

**Fig 3:**
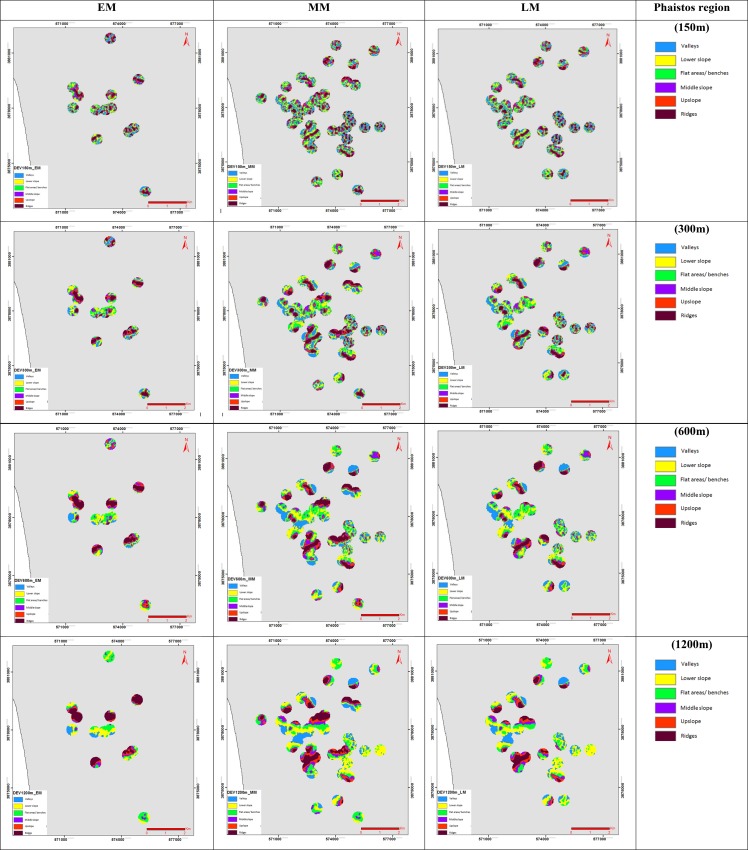
*DEV* for EM, LM and MM period on Phaistos region, with six morphologic classes for the neighbourhood sizes: a) 150 m; b) 300 m; c) 600 m; d) 1200 m (see S2 Fig for Kavousi-Vrokastro region).

The landform classification was based on *TPI* and *DEV* analyses, using as an input dataset the free ASTER Global DEM (G-DEM: 30m pixel size) and the allocated archaeological sites for each Minoan period, as recorded by the archaeological surveys of [6], [33] and [38] (Figs 2 & 3). A buffer zone of 300m around each site was selected for the analyses, in order to evaluate the surrounding dominant landscape of each individual site. The *TPI* and *DEV* analyses were evaluated for various neighbourhood sizes, to select the optimum neighbourhood for the rest of the methodological framework. Such a decision depends on the degree that a particular neighbourhood size will represent, or separate better the various morphological classes in a more coherent and uniform way. The Fragstats free software was used to calculate the patch density (*PD*) and numbers of patches (*NP*), in order to check the fragmentation of the patches for the various morphological classes (Table 1). The *NP* equals to the number of patches of the corresponding patch type, as a measure of the extent of fragmentation of the patch type; and *PD* expresses in addition the number of patches on a per unit area basis [46].

**Table 1:** Indices calculated by using free open source software Fragstats to characterize prior the shape and fragmentation of the patch types.

Indices	Description	Range
***SHAPE***	Equals patch perimeter (m) divided by the square root of patch area (m^2^), adjusted by a constant to adjust for a square standard.	***SHAPE***≦1,without limit.
		***SHAPE*** = 1 when the patch is square and increases without limit as patch shape becomes more irregular.
***PROX***	Equals the sum of patch area (m^2^) divided by the nearest edge-to-edge distance squared (m^2^) between the patch and the focal patch of all patches of the corresponding patch type whose edges are within a specified distance (m) of the focal patch. When the search buffer extends beyond the landscape boundary, only patches contained within the landscape are considered in the computations. Note that the edge-to-edge distances are from cell center to cell center.	***PROX***≥0,
		***PROX*** = 0, if a patch has no neighbors of the same patch type within the specified search radius. ***PROX*** increases as the neighborhood (defined by the specified search radius) is increasingly occupied by patches of the same type and as those patches become closer and more contiguous (or less fragmented) in distribution. The upper limit of ***PROX*** is affected by the search radius and the minimum distance between patches.
***LSI***	Equals .25 (adjustment for raster format) times the sum of the entire landscape boundary (regardless of whether it represents 'true' edge or not, or how the user specifies how to handle boundary/background) and all edge segments (m) within the landscape boundary involving the corresponding patch type, divided by the square root of the total landscape area (m^2^). Note, total landscape area (A) includes any internal background present.	***LSI***≥1,without limit.
		***LSI*** = 1, when landscape consists of a single square patch of the corresponding type and increases without limit as landscape shape becomes more irregular.
***CIRCLE***	Equals 1 minus patch area (m^2^) divided by the area (m^2^) of the smallest circumscribing circle.	0<***CIRCLE***<1,
		***CIRCLE*** approaches 0 for circular patches and approaches 1 for elongated linear patches.
***COHESION***	Equals 1 minus the sum of patch perimeter (in terms of number of cell surfaces) divided by the sum of patch perimeter times the square root of patch area (in terms of number of cells) for patches of the corresponding patch type, divided by 1 minus 1 over the square root of the total number of cells in the landscape, multiplied by 100 to convert to a percentage. Note, total landscape area (Z) excludes any internal background present.	0<***COHESION***<100,
		***COHESION*** approaches 0 as the proportion of the landscape comprised of the focal class decreases and becomes increasingly subdivided and less physically connected. ***COHESION*** increases as the proportion of the landscape comprised of the focal class increases until an asymptote is reached near the percolation threshold.
***PAFRAC***	Equals 2 divided by the slope of regression line obtained by regressing the logarithm of patch area (m^2^) against the logarithm of patch perimeter (m). That is, 2 divided by the coefficient b1 derived from a least squares regression fit to the following equation: ln(area) = b_0_ + b_1_ln(perim). Note, PAFRAC excludes any background patches.	1 ≦ ***PAFRAC*** ≦ 2,
		A fractal dimension greater than 1 for a 2-dimensional landscape mosaic indicates a departure from a Euclidean geometry (i.e., an increase in patch shape complexity). It approaches 1 for shapes with very simple perimeters such as squares, and approaches 2 for shapes with highly convoluted, plane-filling perimeters.
***PD***	Equals the number of patches of the corresponding patch type divided by total landscape area (m^2^)	***PD***>0, constrained by cell size.
		***PD*** is constrained by the grain size of the raster image, because the maximum ***PD*** is attained when every cell is a separate patch. Ultimately cell size will determine the maximum number of patches per unit area
***NP***	Equals the number of patches of the corresponding patch type (class)	***NP***≥1, without limit.
		***NP*** = 1 when the landscape contains only one patch of the corresponding patch type

*TPI* or *DIFF* (z_o_-z) measures the relative topographic position of the central point as the difference between the elevation of this central point and the mean elevation within a pre-determined neighbourhood [30,31]. The result from *TPI* is a positive value when the central point is situated higher than its average neighbourhood, while a negative value indicates a lower location than the average neighbourhood. This index can be used to classify the landscape into morphological classes [47] (Fig 2).

*DEV* ($\frac{zo-z}{SD}$) measures the relative topographic position of the central point as the *TPI* divided by the standard deviation of elevation (*SD*), within a predetermined neighbourhood, where *SD* measures the variability of the cell values in a DEM, around this central point, within the predetermined neighbourhood [30]. The results from *DEV* are: positive, when the central point is situated higher than its average neighbourhood; or negative, when the central point is situated lower than its average neighbourhood (Fig 3).

The exact difference in height between the central point and the average height of the neighbourhood can be highlighted by those analyses, with the range of the outcome values being dependant on the elevation differences in the DEM within the selected neighbourhood. The selection of the neighbourhood sizes were based on 5 out of 12 candidate radii from 100 to 2000m and the ones used to [19] study were decided as optimum for this study. The circular neighbourhood sizes considered within this study as more useful had radii of: 100 m, 300 m, 600 m, 1200 m and 2000 m. The application of a neighbourhood size is important during the analysis and is related to the landscape feature being analysed. In order to classify small features such as streams and valleys a small neighbourhood size need to be used. To identify large canyons or other large landscape features a large neighbourhood size should be selected during the analysis. When we are dealing with larger size of the neighbourhood and large differences in elevation values these conditions result to the wider range of the output values. Based on the above observations *TPI* was used instead of *DEV* for the subsequent analyses of slope position and landform classification, as it represents a more regional representation of the morphological classes (Figs 2 & 3).

### Slope position classification for various neighbourhood sizes

**Fig 4:**
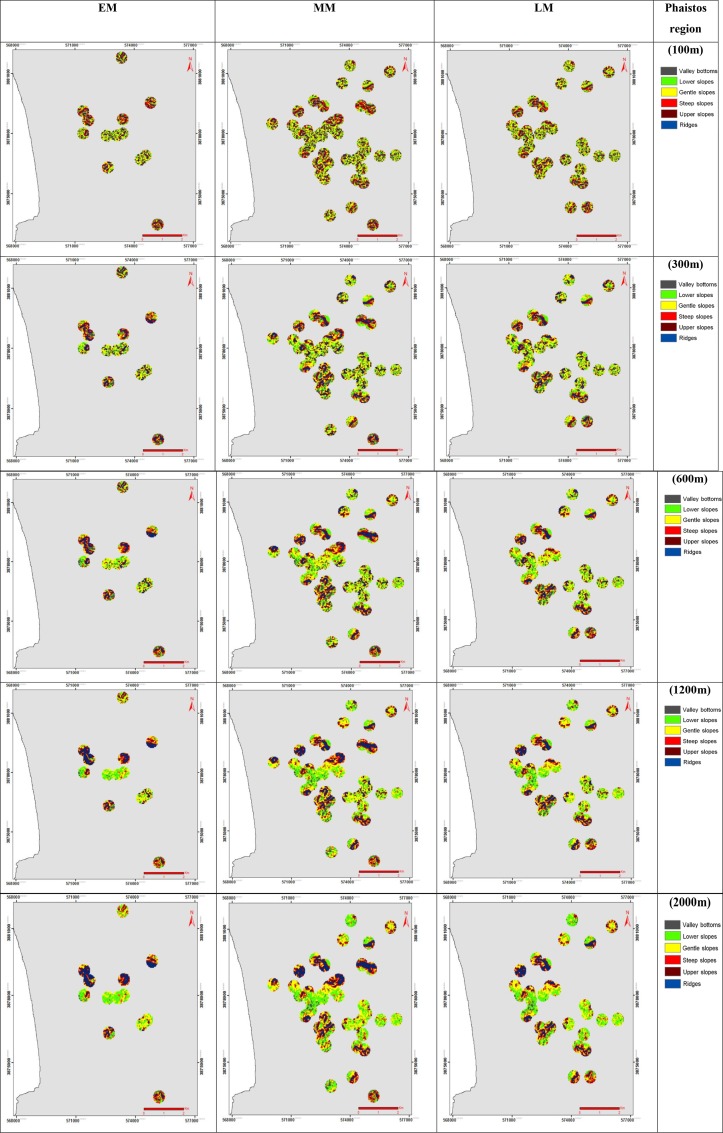
Slope position classification based on *TPI* of the case study sites of Phaistos, for EM, LM and MM periods, with six morphological classes for the neighbourhood sizes: a) 100 m; b) 300 m; c) 600 m; d) 1200 m; e) 2000 m (see S3 Fig for Kavousi-Vrokastro region).

In order to classify the landscape into slope position classes, the variation of *TPI* values by the slope at each point was examined. High *TPI* values determine the tops of the hills, while low *TPI* values highlight valley bottoms. *TPI* values close to 0 are observed over flat to mid-slope areas. In this study, a six-category slope position class (Table 2) was determined for each of the five neighbourhood sizes considered (100 m, 300 m, 600 m, 1200 m and 2000 m) (Fig 4). A 5° slope threshold value was considered for the discrimination of flat areas and mid-slope areas, while a *TPI* threshold value of ±1*SD* was considered for the identification of hilltops and valley bottoms. The six slope position classes, based on [31], are presented in Table 2.

**Table 2:** Classification of the landscape into morphological classes, where *SD* is Standard Deviation.

Morphological classes	Weiss (2001) [31]
Valley bottoms	*TPI*<-*SD*
Lower slopes	-0.5*SD*>*TPI*≥-*SD*
Gentle slopes	0.5*SD* ≥*TPI* ≥-0.5*SD*, slope ≤5^0^
Steep slopes	0.5*SD* ≥*TPI* ≥-0.5*SD*, slope>5^0^
Upper slopes	*SD* ≥*TPI*>0.5*SD*
Ridges	*TPI*>*SD*

### Landform classification for various neighbourhood sizes

**Fig 5:**
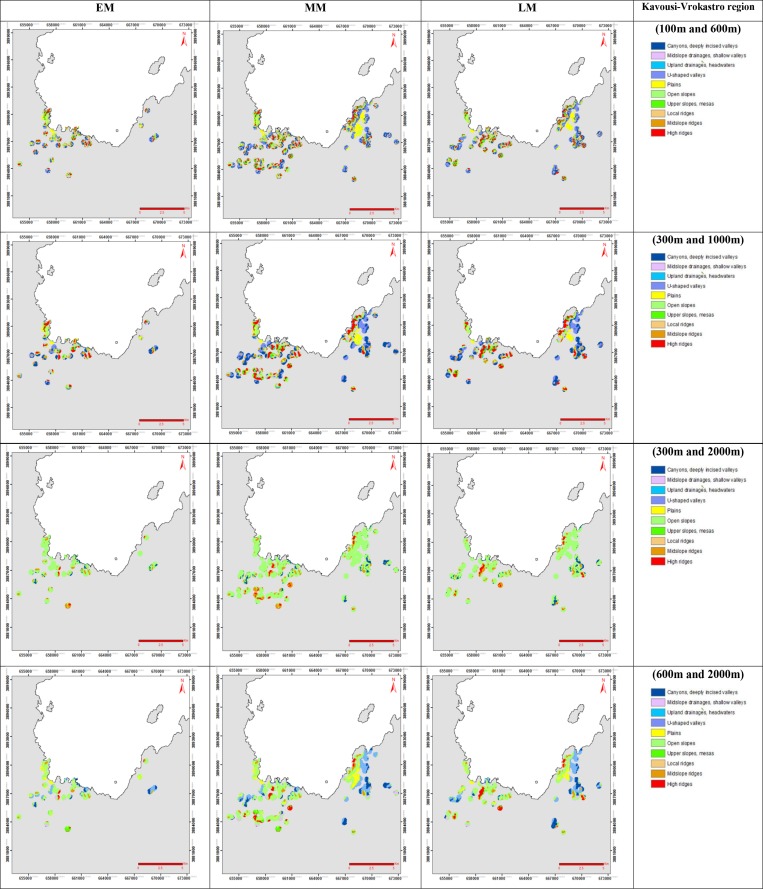
Landform classification based on *TPI* of the case study sites of Kavousi-Vrokastro, for EM, LM and MM periods, with ten landform types for the combined neighbourhood sizes: a) 100 m and 600 m; b) 300 m and 1000 m; c) 300 m and 2000 m; d) 600 m and 2000 m (see S4 Fig for Phaistos region).

The landform classification was based on *TPI* analysis. The *TPI* based landform classification can produce 10 landform classes: streams, mid-slope drainages, local ridges, valleys, plains, foot slopes, upper slopes, upland drainage, mid-slope ridges and high ridges [21] (Fig 5). Usually, two neighbourhood sizes are combined to offer detailed geomorphological information through the discrimination of complex landscape features, as a single neighbourhood size provides less information about the general shape of the landscape [21]. In this study, the two combined neighbourhood sizes in each case were: i) 100 m and 600 m; ii) 300 m and 1000 m; iii) 300 m and 2000 m and; iv) 600 m and 2000 m.

The allocated settlements for each Minoan period in Vrokastro district provided an accuracy assessment of the combined neighbourhood sizes outcomes. Vrokastro district was selected to test the methodology on a more heterogeneous landscape relative to the less rough terrain of Phaistos region. The accuracy assessment consisted of the comparison between the coverage percentage of the individual landforms classes based on *TPI* analysis and the geological-geomorphological description of the settlements provided by the archaeological surveys of [6]. The Fragstats freeware was used to determine which of the combined neighbourhood sizes provided the best representation of the various landform types, relative to the rest of the tested neighbourhood sizes, regarding their shape and fragmentation characteristics. Various indices were examined via Fragstats (Table 1): i) shape index (*SHAPE*); ii) proximity index (*PROX*); iii) landscape shape index (*LSI*); iv) related circumscribing circle (*CIRCLE*); v) patch cohesion index (*COHESION*); vi) patch density (*PD*); vii) numbers of patches (*NP*) and; viii) perimeter-area fractal dimension (*PAFRAC*).

A spatial autocorrelation evaluation based on Moran’s I approach was carried out for the EM, MM, LM settlements and 100 random sites of the study area, to check whether the pattern expressed was clustered or random [48]. The derived z‐score or p‐value can indicate statistical significance, with a positive Moran's I index value indicating tendency toward clustering, while a negative Moran's I index value indicates tendency toward dispersion. The z‐score and p‐value calculations indicate whether the null hypothesis can be rejected. In this case, the null hypothesis states that features are randomly distributed across the study area. Clustering is a statistical classification technique for separating spatial information into relatively homogeneous groups: similarities are high between members belonging to a class, or cluster [48].

## Results

### *TPI* and *DEV* analyses for various neighbourhood sizes

The *TPI* and *DEV* outcomes are presented on Figs 2 and 3 for the various neighbourhood sizes (150 m, 300 m, 600 m, 1200 m and 2000 m), with six slope classes being discriminated (Table 3). The results show that *TPI* provides a generalised representation of topography, while *DEV* highlights subtle topographic variations, which is in accordance with the findings of [32]. This study focuses on the regional representation of the morphological classes so the generalised approach of *TPI* was selected, instead of *DEV*, for the analyses of slope position and landform classification evaluation. The calculated indices of *NP* and *PD*, revealed that *DEV* provides a more fragmented representation of the morphological classes than *TPI*, with the *PD* values indicating a higher density of patches for the individual classes (Table 3). The comparison of the *NP* and *PD* values between the various neighbourhood sizes, for both *DEV* and *TPI*, indicated that the 150 m neighbourhood size shows high fragmentation with a large number of patches and high density of patches. On the other hand, the 600 m and 1200 m neighbourhood sizes highlight a uniform composition of the morphological classes (steady decrease of *NP* and *PD*), which results to a high degree of generalization regarding the morphological classes, with particular features not visible at that scale, due to the generalization (Table 3). As a result, the 300m neighbourhood size has been selected as optimum for the rest of our analyses: it discriminates the various features with less fragmentation and diverse *NP* and *PD* values, without a high degree of generalization.

**Table 3:** Comparison of various neighbourhood sizes between *DEV* and *TPI* regarding fragmentation, to identify which neighbourhood size better highlights small or large geomorphological features.

Morphological Classes	DEV (150m)		DEV (300m)		DEV (600m)		DEV (1200m)	
	***NP***	***PD***	***NP***	***PD***	***NP***	***PD***	***NP***	***PD***
**Ridge**	630	17.98	286	8.16	161	4.59	113	3.22
**Upper slope**	1416	40.43	520	14.84	252	7.19	122	3.48
**Middle slope**	1483	42.34	586	16.73	245	6.99	148	4.22
**Flat area/ bench**	1559	44.51	539	15.38	249	7.10	119	3.39
**Lower slope**	1330	37.97	483	13.79	190	5.42	88	2.51
**Valley**	510	14.56	202	5.76	90	2.56	49	1.39
**Morphological Classes**	**TPI (150m)**		**TPI (300m)**		**TPI (600m)**		**TPI (1200m)**	
	***NP***	***PD***	***NP***	***PD***	***NP***	***PD***	***NP***	***PD***
**Ridge**	335	9.57	154	4.43	81	2.31	49	1.40
**Upper slope**	935	26.71	340	9.71	172	4.91	98	2.80
**Middle slope**	933	26.65	393	11.22	181	5.17	111	3.17
**Flat area/ bench**	1137	32.48	439	12.54	233	6.65	109	3.11
**Lower slope**	885	25.28	344	9.82	141	4.02	76	2.17
**Valley**	312	8.91	139	3.97	79	2.25	34	0.97

### Slope position classification for various neighbourhood sizes

Slope position classification based on *TPI* for Early, Middle and Late Minoan periods consisted of six classes (Fig 4). The percentages of the slope position classification areal coverage, for the individual Minoan periods, are presented on Table 4. For valley bottoms, a decrease in the coverage percentage exists with increasing neighbourhood sizes, for all Minoan periods. For lower slopes, there is an increase of coverage with a neighbourhood size increase, for all Minoan periods. Gentle slopes, steep slopes and ridges have a steady variable coverage percentage for all Minoan periods. The two notable observations on those landform types are that: i) the upper slopes reveal a high decrease between 100 m and 300 m neighbourhood sizes, for all Minoan periods, but then a steady variation exists; ii) during the Early Minoan period, the ridges class coverage increases gradually with increased neighbourhood sizes; while during the Middle and Late Minoan periods there is a steady variation of ridges class coverage between the neighbourhood sizes. This might be a result that during Early Minoan period the settlements were developed on high hilltops, presumably for defensive purposes, so they were located on rougher terrain relative to Middle and Late Minoan. The neighbourhood size of 600m provides the better option to characterize the region, based on the indices statistics, visual interpretation of satellite imagery, in conjunction with information from the derived geomorphometric variables and the landscape shape/fragmentation indices (*NP*, *PD*, *PROX* and *LSI*) (Fig 4; Tables 4 & 5). The *NP* and *PD* indices indicate that lower neighbourhood size represents a fragmented representation of the morphological classes, with a higher density of patches for the individual classes. As the neighbourhood size increases, a uniform composition of the morphological classes (steady decrease of *NP* and *PD*) is observed. These indices, in conjunction with the rest of the indices, imply that the 600 m neighbourhood size is the optimum neighbourhood (Table 5). This particular neighbourhood size defines better the morphological classes, giving equal emphasis on regional and subtle topography, without exaggerating one or other morphological classes as it happens with the rest of the neighbourhood sizes.

**Table 4:** Slope position classes areal coverage (%) of the case study sites, for the Early, Middle and Late Minoan periods, regarding the six morphological classes for the selected neighbourhood sizes.

**Slope position classes**	**Areal coverage (%) for the individual neighbourhood sizes —Early Minoan period**				
	**Neighbourhood size: 100 m**	**Neighbourhood size: 300 m**	**Neighbourhood size: 600 m**	**Neighbourhood size: 1200 m**	**Neighbourhood size: 2000 m**
Valley bottoms	11.62	11.68	9.38	7.06	6.03
Lower slopes	18.45	18.84	19.14	21.03	21.4
Gentle slopes	14.75	15.22	15.97	15.44	15.88
Steep slopes	26.53	24.49	25.01	24.94	25.22
Upper slopes	15.02	13.18	13.23	13.42	12.88
Ridges	13.63	16.59	17.27	18.11	18.6
**Slope position classes**	**Areal coverage (%) for the individual neighbourhood sizes —Middle Minoan period**				
	**Neighbourhood size: 100 m**	**Neighbourhood size: 300 m**	**Neighbourhood size: 600 m**	**Neighbourhood size: 1200 m**	**Neighbourhood size: 2000 m**
Valley bottoms	11.93	11.23	10.09	9.38	7.96
Lower slopes	18.95	22.27	24.49	26.53	28.09
Gentle slopes	17.19	17.55	17.33	16.03	15.43
Steep slopes	24.51	23.43	23.9	23.86	23.67
Upper slopes	14.29	11.89	11.37	11.09	10.84
Ridges	13.14	13.64	12.82	13.12	14.01
**Slope position classes**	**Areal coverage (%) for the individual neighbourhood sizes —Late Minoan period**				
	**Neighbourhood size: 100 m**	**Neighbourhood size: 300 m**	**Neighbourhood size: 600 m**	**Neighbourhood size: 1200 m**	**Neighbourhood size: 2000 m**
Valley bottoms	11.79	10.81	10.34	9.64	7.5
Lower slopes	19.12	23.18	25.0	26.65	28.23
Gentle slopes	17.83	17.98	17.59	16.19	15.71
Steep slopes	24.25	23.27	23.88	23.59	23.54
Upper slopes	14.21	11.53	11.11	11.19	11.13
Ridges	12.8	13.23	12.09	12.74	13.89

**Table 5:** Indices used for the evaluation of the slope classification for the various neighbourhood sizes and the selection of the optimum, regarding their shape and fragmentation characteristics.

**Slope position classes**		**Neighbourhood size:100m**						
	***NP***	***PD***	***LSI***	***SHAPE***	***CIRCLE***	***PAFRAC***	***PROX***	***COHESION***
**Valley bottoms**	1757	50.2	51.15	1.18	0.47	1.36	1.09	65.43
**Lower slopes**	3028	86.51	77.9	1.325	0.41	1.59	3.4	70.88
**Gentle slopes**	1503	42.94	57.6	1.329	0.42	1.52	9.26	84.5
**Steep slopes**	1979	56.54	79.5	1.51	0.49	1.66	16.38	87.15
**Upper ridges**	3234	92.4	75.97	1.25	0.39	1.6	2	62.07
**Ridges**	1295	37	47.69	1.27	0.54	1.4	1.37	72.5
**Slope position classes**		**Neighbourhood size:300m**						
	***NP***	***PD***	***LSI***	***SHAPE***	***CIRCLE***	***PAFRAC***	***PROX***	***COHESION***
**Valley bottoms**	1097	31.34	39.35	1.22	0.46	1.33	1.84	75.16
**Lower slopes**	1722	49.2	64.04	1.44	0.46	1.56	7.66	83.4
**Gentle slopes**	1297	37.05	50.13	1.32	0.43	1.46	10.68	87.58
**Steep slopes**	1448	41.37	72.31	1.58	0.47	1.64	16.34	89.02
**Upper ridges**	2097	59.91	62.98	1.29	0.42	1.57	2.63	69.21
**Ridges**	618	17.65	34.21	1.35	0.55	1.36	3.26	84.7
**Slope position classes**		**Neighbourhood size:600m**						
	***NP***	***PD***	***LSI***	***SHAPE***	***CIRCLE***	***PAFRAC***	***PROX***	***COHESION***
**Valley bottoms**	907	25.91	33.92	1.2	0.42	1.33	2.24	79.3
**Lower slopes**	1184	33.83	56.61	1.52	0.49	1.55	15.13	89.5
**Gentle slopes**	1254	35.83	48.09	1.31	0.43	1.46	10.04	87.86
**Steep slopes**	1236	35.31	68.53	1.62	0.5	1.63	23.61	91.59
**Upper ridges**	1602	45.77	56.53	1.32	0.43	1.57	3.29	74.47
**Ridges**	465	13.28	29.5	1.37	0.54	1.36	5.52	88.54
**Slope position classes**		**Neighbourhood size:1200m**						
	***NP***	***PD***	***LSI***	***SHAPE***	***CIRCLE***	***PAFRAC***	***PROX***	***COHESION***
**Valley bottoms**	727	20.77	29.65	1.2	0.39	1.33	3.66	82.87
**Lower slopes**	931	26.6	48.89	1.49	0.47	1.53	20.96	93.89
**Gentle slopes**	1246	35.6	46.51	1.3	0.44	1.45	8.62	86.08
**Steep slopes**	1137	32.48	65.53	1.62	0.49	1.61	21.52	91.66
**Upper ridges**	1269	36.25	52.61	1.36	0.45	1.56	4.69	80.27
**Ridges**	371	10.6	26.18	1.39	0.56	1.35	8.39	90.11
**Slope position classes**		**Neighbourhood size:2000m**						
	***NP***	***PD***	***LSI***	***SHAPE***	***CIRCLE***	***PAFRAC***	***PROX***	***COHESION***
**Valley bottoms**	671	19.17	27.92	1.19	0.39	1.31	2.5	80.71
**Lower slopes**	828	23.65	42.9	1.45	0.46	1.5	21.71	94.29
**Gentle slopes**	1211	34.6	44.82	1.28	0.42	1.45	8.07	86.79
**Steep slopes**	1061	30.31	63.15	1.64	0.51	1.6	25.2	91.89
**Upper ridges**	1180	33.71	50.76	1.36	0.43	1.56	4.98	80.67
**Ridges**	328	9.37	24.9	1.42	0.57	1.35	11.69	91.23

### Landforms classification for various neighbourhood sizes

**Fig 6:**
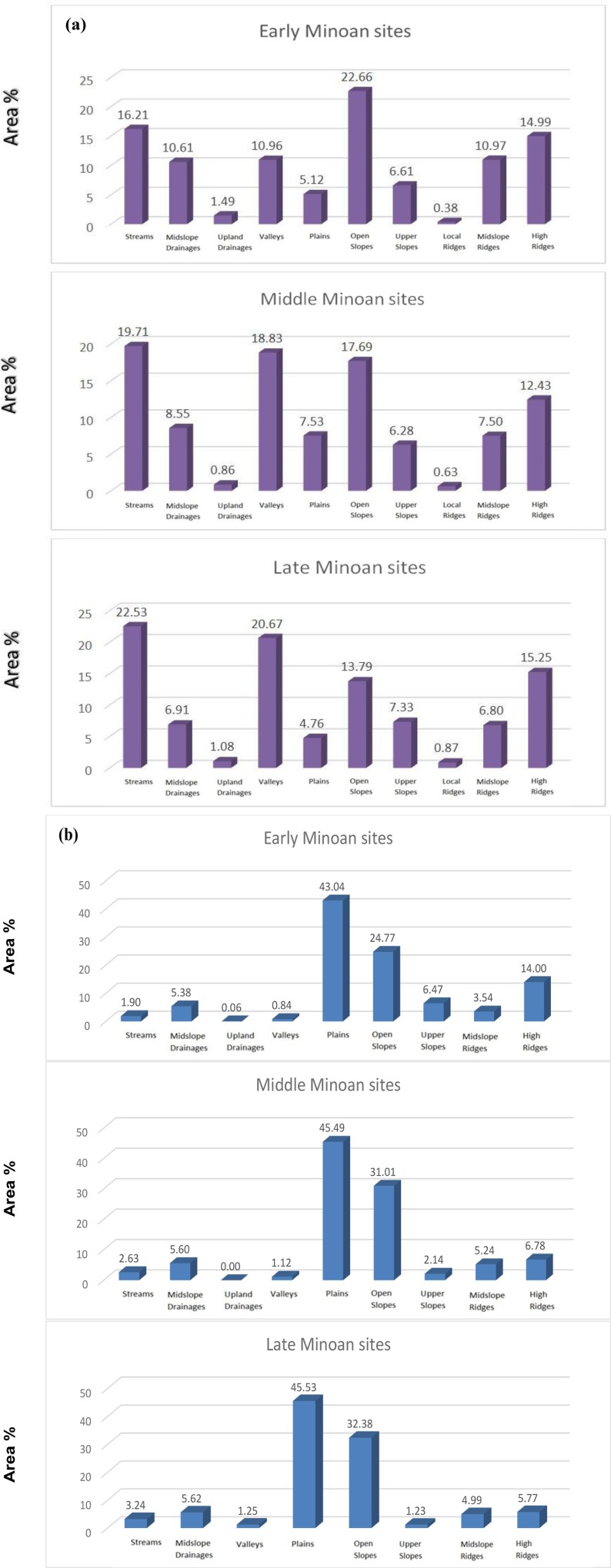
Histogram of landform elements (%) for the combined neighbourhood sizes 300 m and 1000 m. (a) Kavousi- Vrokastro case study area; (b) Phaistos case study area.

The *TPI* based landform classification for the Early, Middle and Late Minoan periods consisted of ten different landform types, for combined neighbourhood sizes (Fig 5). The combined neighbourhood sizes of 300 m and 1000 m were optimal for characterizing the region, based on the Fragstats indices statistics and visual interpretation of satellite imagery (Table 6). Moreover, the study areas are located on rough terrain with abrupt changes of the topography, so a more generalised overview from the combined neighbourhood sizes of 300 m and 1000 m is considered as optimum for larger scale geo-archaeological interpretations. The more subtle topography determined by the combined neighbourhood sizes of 100 m and 600 m would be suitable for flat areas/benches, with the landform types being less fragmented. The histogram of landform elements percentage for the combined neighbourhood sizes of 300 m and 1000 m reveals the predominant landform types during each of the Minoan periods (Fig 6).

**Table 6:** Indices being used for the evaluation of the landform classification for the various combined neighbourhood size and the selection of the optimum neighbourhood, regarding their shape and fragmentation characteristics.

**Morphological Classes**	**Combined neighbourhood sizes: 100m and 600m**							
	***NP***	***PD***	***LSI***	***SHAPE***	***CIRCLE***	***PAFRAC***	***PROX***	***COHESION***
**Canyons, deeply incised valleys**	292	8.34	23.88	1.4	0.53	1.337	7.29	89.7
**Midslope drainages,shallow valleys**	526	15.02	28.17	1.25	0.46	1.331	1.45	79.7
**Upland drainages**	132	3.77	13.61	1.21	0.43	1.31	0.69	71.8
**U-shaped valleys**	319	9.11	26.33	1.53	0.48	1.43	14.46	92.55
**Plains**	277	7.91	19.81	1.33	0.44	1.34	46.25	96.51
**Open slopes**	621	17.74	41.88	1.56	0.49	1.48	16.59	92.25
**Upper slopes, mesas**	231	6.6	24.97	1.58	0.5	1.45	9.51	89.51
**Local ridges**	166	4.74	15.37	1.22	0.46	1.3	0.97	74.83
**Midslope ridges**	522	14.91	27.28	1.23	0.45	77.82	1.31	1.30
**High ridges**	193	5.51	19.12	1.39	0.54	1.29	8.69	90.21
**Morphological Classes**	**Combined neighbourhood sizes: 300m and 1000m**							
	***NP***	***PD***	***LSI***	***SHAPE***	***CIRCLE***	***PAFRAC***	***PROX***	***COHESION***
**Canyons, deeply incised valleys**	122	3.48	14.45	1.44	0.527	1.23	15.42	94.22
**Midslope drainages,shallow valleys**	178	5.08	16.98	1.39	0.48	1.29	1.72	88.89
**Upland drainages**	34	0.97	6.62	1.23	0.42	1.27	1.41	83.53
**U-shaped valleys**	153	4.37	16.3	1.53	0.526	1.32	11.93	94.1
**Plains**	230	6.57	18.65	1.35	0.44	1.35	43.35	96.41
**Open slopes**	426	12.17	31.78	1.53	0.51	1.4	15.62	92.61
**Upper slopes, mesas**	126	3.6	15.6	1.47	0.49	1.35	6.68	90.71
**Local ridges**	49	1.4	8.7	1.33	0.45	1.39	0.74	75.27
**Midslope ridges**	179	5.11	16.48	1.34	0.45	1.25	2.58	88.69
**High ridges**	91	2.6	11.86	1.37	0.54	1.19	9.82	94.13
**Morphological Classes**	**Combined neighbourhood sizes: 300m and 2000m**							
	***NP***	***PD***	***LSI***	***SHAPE***	***CIRCLE***	***PAFRAC***	***PROX***	***COHESION***
**Canyons, deeply incised valleys**	68	1.94	10.73	1.42	0.54	1.26	2.63	91.04
**Midslope drainages,shallow valleys**	11	0.31	4.37	1.41	0.53	1.22	0	87.95
**Upland drainages**	-	-	-	-	-	-	-	-
**U-shaped valleys**	-	-	-	-	-	-	-	-
**Plains**	153	4.37	16.7	1.4	0.45	1.36	64.81	96.53
**Open slopes**	247	7.05	19.51	1.54	0.481	1.27	55.08	97.53
**Upper slopes, mesas**	2	0.05	2.14	1.5	0.71	N/A	2.87	79.46
**Local ridges**	-	-	-	-	-	-	-	-
**Midslope ridges**	162	4.62	15.61	1.34	0.488	1.22	7.06	91.14
**High ridges**	38	1.08	7.19	1.28	0.53	1.16	2.32	89.57
**Morphological Classes**	**Combined neighbourhood sizes: 600 m and 2000 m**							
	***NP***	***PD***	***LSI***	***SHAPE***	***CIRCLE***	***PAFRAC***	***PROX***	***COHESION***
**Canyons, deeply incised valleys**	215	6.14	19.10	1.41	0.48	1.31	49.75	95.97
**Midslope drainages,shallow valleys**	59	1.68	10.62	1.49	0.54	1.27	5.15	92
**Upland drainages**	-	-	-	-	-	-	-	-
**U-shaped valleys**	38	1.08	9.08	1.68	0.55	1.25	46.59	97.1
**Plains**	215	6.14	16.15	1.28	0.47	1.35	9.75	93.66
**Open slopes**	306	8.74	24.64	1.55	0.5	1.32	55.06	96.6
**Upper slopes, mesas**	39	1.11	7.6	1.39	0.49	1.23	6.73	92.01
**Local ridges**	2	0.05	2.57	1.8	0.8	N/A	0	79.63
**Midslope ridges**	62	1.77	9.52	1.36	0.46	1.22	6.79	91.41
**High ridges**	30	0.85	6.66	1.35	0.5	1.17	5.47	92.5

For the Kavousi-Vrokastro district, two main highlights of the settlements location, from the Early Minoan towards the Late Minoan are: i) high increase on deeply incised streams, valleys and plains; ii) high decrease on open slopes, mid-slope drainages and mid-slope ridges (Fig 6). The settlement types and the number of settlements are presented in Table 7. For the Phaistos district, two main highlights of the settlements location from the Early to the Late Minoan are: i) decrease of high ridge and upper slope locations; ii) steady increase of locations in deeply incised valleys, plains and open slopes (Fig 6). The Phaistos district has less rough terrain than Kavousi-Vrokastro, so the tendency of populations moving to flatter landscape from the Early to Late Minoan, is more pronounced. On the other hand, there is a drop in the number of settlements found on high ridges, which indicates a tendency towards abandoning high-elevation settlements. These interpretations are in accordance with findings from the archaeological surveys of [6,33,38]. Based on Moran’s I analysis of the EM, MM, LM settlements and 100 random sites to check whether the pattern expressed is clustered or random, the outcomes from the Kavousi-Vrokastro study showed the following case study: i) for the 100 random points the z‐score was -0,75 and p‐value 0.45, indicating that the pattern is random; ii) similar observations exist for the EM settlements with the z‐score being 1.05 and p‐value 0.29. However, for the MM settlements the z‐score was 7,76 and p‐value 0, indicating that there is less than 1% likehood that this clustered pattern could be a result of random chance. Furthermore, with LM settlements the z‐score was 3.15 and p‐value 0,001, indicating a clustered pattern. Similar observations also exist for the Phaistos case study: i) for the 100 random points the z‐score was 1.47 and the p‐value was 0.14, indicating that the pattern does not appear to be significantly different than random; ii) similarly random pattern exists for the EM settlements with the z‐score being -0.98 and the p‐value being 0.32. On the other hand, for the MM settlements the z‐score was 5 and the p‐value was 0, indicating that there is less than 1% likehood that this clustered pattern could be a result of random chance; while for the LM settlements the z‐score was 2.84 and the p‐value was 0,0044, indicating a clustered pattern.

**Table 7:** Settlement types and number of settlements occurring over specific landform types.

Kavousi/Vrokastro	*Early Minoan*	*Middle Minoan*	*Late Minoan*
*Farmhouse*	*0*	*31*	*7*
*Village*	*42*	*103*	*76*
*Household*	*0*	*2*	*3*
*Palace*	*0*	*1*	*0*
Total	42	137	86
**Landform types:**			
Mid-slope, upper slopes, high ridges	19 (out of total)	40 (out of total)	31 (out of total)
Plains, open slopes	17 (out of total)	35 (out of total)	23 (out of total)

## Discussion

This study has quantified the spatio-temporal variations of Early, Middle and Late Minoan settlement locations in the landscapes of Crete. A general trend is observed during the Early to Middle Minoan, with the population moving inland in search of arable land, most settlements initially being located within 1.5 km of the coast. During the Middle Minoan, there was an increase of settlements over low-gradient slopes, upper slopes and mesas in hilly terrain, where they remained during the Late Minoan, probably because of their strong defensive advantage (Fig 6).

The analysis of settlement areas indicates that during the Middle Minoan there was: i) an increase of settlements in deeply incised valleys, with people looking for access to perennial water supplies and; ii) a reduction of the number of settlements in shallow U-shaped valleys, mild slopes & mid-slope ridges, perhaps due to movement of population to more arable land on the plains and U-shaped valley floors (Fig 6) [6,33,38].

In Phaistos district, the Early Minoan settlements are located in heterogeneous landscapes, distributed sparsely over variable terrain. In the Middle Minoan the settlements are found over homogeneous landscapes, such as the plains. In the Late Minoan the largest settlements are found on the plains and lowlands. During the Middle to Late Minoan, the number of settlements decreased, especially at higher elevations; the settlements on the plains remained, while farming increased and population concentrated in the larger settlements [6,33,38].Based on the analysis of the area percentage of the Minoan settlements in Phaistos district, population increased within the deeply incised valleys, perhaps due to the need for perennial water, and on the plains and foot slopes, perhaps due to increased arable land access. However, population decreased on the upper slopes, mesas and high ridges, perhaps as a result of the observed movement to arable lowlands (Fig 6).

The accuracy assessment, comparing the findings of this study with the archaeological surveys of [6] (Table 8), shows a low percentage of no agreement (14%) while there is a substantial percentage of agreement (64% of moderate agreement and 22% of high agreement). That implies that the rapid low-cost geoinformatic techniques used in this study are a cost-effective technique that can complement conventional archaeological surveys.

**Table 8:** Accuracy assessment, whether there is an agreement or not, comparing the coverage percentage of the individual landforms classes, based on *TPI*, and the settlement geological-geomorphological description provided from the archaeological surveys of Hayden et al. (2004). The qualitative agreement type was based on the overall areal percentage coverage of associated landform types: Low (<50%), Moderate (50–70%) and High (>70%).

*Early Minoan*	Hayden et al., 2004 [6]	Landforms classification based on *TPI*	Agreement
*Phrouzi area*	Complex geology of marly limestone, graniodorite with settlements found on Kedromouri ridges. Series of hills, ridges and plateaus. **Sites ID: KM1-3**	**KM 1**: **55. 5%** coverage from high ridges and upper slopes.	**Moderate**
		**KM 2**: **32%** coverage from high ridges.	**Low**
		**KM 3**: **36%** coverage from high ridges and mid-slope ridges	**Low**
*Phanourios area*	Lies on a broad plateau. **Site ID:Aph1**	**Aph1: 56%** coverage from open slopes, plains and upper slopes, mesas	**Moderate**
*Istron river valley*	Slope of 20^0^−30^0^, steep and eroded bedrock slope. **Site ID: GN2A:2**	**GN2A:2: 68%** coverage from high ridges, open slopes and mid-slope ridges	**Moderate**
*Vrokastro basin*	Located on a bluff behind steep slope and cliff overlooking the Xeropotamos river. **Site ID: PT1**	**PT1: 47%** coverage from local ridges, open slopes and mid-slope ridges	**Low**
***Middle Minoan***	**Hayden et al., 2004 [6]**	**Landforms classification based on *TPI***	**Agreement**
*Meseleroi valley*	Meseleroi valley is an area with complex geology. Sites lie on slopes intersected with brecciated limestone and dark gray limestone, flanked by higher slopes of conglomerate. Close to perennial springs. **Sites ID: OL8, OL12**	**OL8: 52%** coverage from deeply incised valleys.	**Moderate**
		**OL12: 61%** coverage from deeply incised valleys and high ridges.	**Moderate**
*Spilia/Kalives*, *Xivouni and Katsikadara*	Occupation continues and expands on the conglomerate hills and slopes encircling the Istron valley. **Sites ID: SP1A,SP2,SP3,KK1,KK5,KK6,PI1**	**SP1A: 43%** coverage from deeply incised valleys, mid-slope ridges and local ridges	**Low**
		**SP2: 53.5%** coverage from deeply incised valleys.	**Moderate**
		**SP3: 46.5%** coverage from deeply incised valleys.	**Low**
		**KK1: 62%** coverage from deeply incised valleys.	**Moderate**
		**KK5: 63%** coverage from deeply incised valleys.	**Moderate**
		**KK6: 75%** coverage from deeply incised valleys and mid-slope ridges.	**High**
		**PI1: 63%** coverage from deeply incised valleys.	**Moderate**
*Ioannimiti region*	These sites are located on the hilltops, slopes and ridges. **IM1** located on an east facing rise, **IM2** lies along the western side of the Vathi peak, with excellent view of Ioannimiti area. Terrace walls constructed of large boulders still stand on the gentle, 15^0^, south facing slopes where **IM3** is located. **IM9** on a low south facing slope above a torrent bed at the centre of the promontory. **Sites ID: IM1, IM2, IM3 IM9**	**IM1: 67%** coverage from open slopes.	**Moderate**
		**IM2: 39%** coverage from high ridges.	**Low**
		**IM3: 54%** coverage from open slopes and deeply incised valleys.	**Moderate**
		**IM9: 49%** coverage from open slopes.	**Low**
*Ioannimiti region*	Gentle slope, east facing slope. **Site ID: IM13**	**IM13: 76%** coverage from open slopes.	**High**
*Prophitis Ilias*, *Tsigouni*, *Tzamachi*	Exploitation of the landscape occurred on a large-scale in upland, inland areas not extensively settled until this period. These regions have an elevational range between 450–700 m asl and comprise small basins and naturally terraced slopes, with dominant formations being the conglomerates and marl soils. **Sites ID: PI2,PI3,PI5,TS1,TM1,TM2,TM3, TM4,TM8**	**PI2: 51.5%** coverage from open slopes and mid-slope drainages.	**Moderate**
		**PI3: 53.5%** coverage from open slopes and high ridges.	**Moderate**
		**PI5: 83%** coverage from high ridges and upper slopes, mesas.	**High**
		**TS1: 73.5%** coverage from high ridges and upper slopes, mesas.	**High**
		**TM1: 78%** coverage from open slopes and mid-slope drainages.	**High**
		**TM2: 50%** coverage from open slopes and mid-slope ridges.	**Moderate**
		**TM3: 51.5%** coverage from open slopes.	**Moderate**
		**TM4: 62%** coverage from deeply incised valleys.	**Moderate**
		**TM8: 65%** coverage from open slopes and high ridges.	**Moderate**
*Elias to Nisi and Kopranes region*	New sites located on the promontories of Miocene white or gray marls (i.e. Elias to Nisi) which extend south to the lower base of hills that form Kopranes. **KP1** is located higher up the slopes of Kopranes. **Sites ID: EN3,KP1**	**EN3: 59.5%** coverage from U-shaped valleys and open slopes.	**Moderate**
		**KP1: 59%** coverage from U-shaped valleys and deeply incised valleys.	**Moderate**
*Agios Phanourios and Vrokastro region*	**APh3** the largest and most important settlement in the region. It is located on a long, 10°–20°, north-facing slope that ascends south to a ridge The top of the ridge extends west to the area immediately south of the Vrokastro peak and the Vrokastro settlement. **VK1. Sites ID: APh3, VK1**	**APh3: 67.5%** coverage from upper slopes, mesas and high ridges.	**Moderate**
		**VK1: 74%** coverage from high ridges and upper slopes, mesas.	**High**
*Prina region*	**PN1** was the first settlement noted on EM period on the peak of Stavros continuing into MM period, with **PN4** site found nearby (400m east of the peak), suggesting habitation on this slope with evidence of cooking, storage and fine wares of MM period. **Sites ID: PN1, PN4**	**PN1: 50%** coverage from mid-slope ridges and open slopes.	**Moderate**
		**PN4: 83%** coverage from U-shaped valleys and deeply incised valleys.	**High**
**Late Minoan**	**Hayden et al., 2004 [6]**	**Landforms classification based on *TPI***	**Agreement**
Istron valley and Prophitis Ilias	Pottery of LM period on site **KK5**, southern end of Istron valley, flanked to the east by steep slopes that go up to Prophitis Ilias (**PI3,PI5,PI6**); comprises the upper slopes of the gorge linking the Istron valley to Prina, Meseleroi valley and Prophitis Ilias basin. **Sites ID: KK5, PI3,PI5,PI6**	**KK5: 62%** coverage from deeply incised valleys	**Moderate**
		**PI3: 65%** coverage from open slopes, upper slopes, mesas and high ridges	**Moderate**
		**PI5: 82%** coverage from upper slopes, mesas and high ridges	**High**
		**PI6: 65%** coverage from high ridges	**Moderate**
Aphendi Christos valley	**AC2** located on the lower eastern slopes of the Aphendi Christos valley was buried to a depth of 0.50–1m. **Site ID: AC2**	**AC2: 66%** coverage from deeply incised valleys and U-shaped valleys.	**Moderate**
Kendromouri hills	Occupation continued in the Kendromouri hills; LM pottery on sites **KM1, KM2. KM2** is Neopalatial and the ridge was used for settlement during many phases. A massively built structure terraced into the southwestern slopes of the ridge could be a Neopalatial farmstead based on associated pottery. **Sites ID: KM1,KM2**	**KM1: 60%** coverage from high ridges and open slopes	**Moderate**
		**KM2: 50%** coverage from high ridges and open slopes	**Moderate**
Istron river valley	**GN1:2**, lies at the south-eastern base of a hill. A built tomb exists, which indicates the presence of a habitation, based on the lower slopes of this hill. A large Neopalatial farmstead, **SP2**, was identified on the western slopes at the mouth of deep Katsidara gorge. It flanks the upper route, a calderimi, through the gorge. On the eastern side of the valley a site (**KK7**) continues into the Neopalatial period on a long ridge flanking the west side of the Aphendi Christos valley. The steep slopes of this area have been terraced and are suited for cultivation. **Sites ID: GN1:2, SP2, KK7**	**GN1:2: 67%** coverage from U-shaped valleys and open slopes.	**Moderate**
		**SP2: 52.3%** coverage from deeply incised valleys	**Moderate**
		**KK7: 63%** coverage from open slopes, mid-slope drainages and mid-slope ridges	**Moderate**
Vrokastro and Agios Phanourios	Strong evidence for continuity of settlements from the Neopalatial period exists in an area of rolling hill, plateaus and ravines south of the summit of Vrokastro. **APh3** is the primary settlement with ancillary sites to the south (**APh2,APh10**). The Neopalatial site **VK5**, on the ridge top overlooking Aphendi Christos valley to the south of Vrokastro summit. **DL1** lies on the north-facing slopes of the Duo Laggadia ridge. **Sites ID: APh3,APh2,APh10,VK5,DL1**	**APh3: 65%** coverage from upper slopes, mesas and open slopes	**Moderate**
		**APh2: 88%** coverage from upper slopes, mesas and high ridges	**High**
		**APh10: 63%** coverage from upper slopes, mesas and high ridges	**Moderate**
		**VK5: 78%** coverage from upper slopes, mesas and high ridges	**High**
		**DL1: 72%** coverage from mid-slope ridges and open slopes	**High**

## Conclusion

During the last decade the quantification of landform types has attracted the interest of the geoinformatic research community, with various studies using GIS-based approaches. The evaluation of geomorphometric datasets can be integrated with GIS techniques, highlighting the information within the interlinked geographic data. The extracted information can be particularly useful for landform classification, as this case study of Crete has illustrated. Such information is useful when linked to geospatial data about the distributions of archaeological sites over space and time.

This investigation of Phaistos, Kavousi and Vrokastro districts has produced valuable information regarding the distribution of settlements during the Early, Middle and Late Minoan periods. Based on the analysis of this study, a general trend is observed during the Early to Middle Minoan, with population moving from heterogeneous terrain at higher elevation, to the lowlands (Fig 6). During the Middle to Late Minoan, the population remained in the arable lowlands, with better organization and concentration in larger settlements.

This study was constrained by the limited amount of paleo-environmental data available for Crete, resulting in a knowledge gap that adds to the uncertainty associated with the interpretations made about the factors driving the variations in the Minoan settlement distributions. Nevertheless, the methodology presented here can provide useful spatio-temporal analyses, at district scales, for future studies to examine at local scales, with associated studies of palaeo-environmental conditions or archaeological predictive modelling. In addition, this study offers valuable information for further research, where socio-economic or political factors can be considered for settlement hierarchy assessments.

## Supporting information

S1 Fig *TPI or DIFF* for EM, LM and MM period on Kavousi-Vrokastro region, with six morphologic classes for the neighbourhood sizes: a) 150 m; b) 300 m; c) 600 m; d) 1200 m.
(TIF)

S2 Fig *DEV* for EM, LM and MM period on Kavousi-Vrokastro region, with six morphologic classes for the neighbourhood sizes: a) 150 m; b) 300 m; c) 600 m; d) 1200 m.
(TIF)

S3 Fig Slope position classification based on *TPI* of the case study sites of Kavousi-Vrokastro, for EM, LM and MM periods, with six morphological classes for the neighbourhood sizes: a) 100 m; b) 300 m; c) 600 m; d) 1200 m; e) 2000 m.
(TIF)

S4 Fig Landform classification based on *TPI* of the case study sites of Phaistos, for EM, LM and MM periods, with ten landform types for the combined neighbourhood sizes: a) 100 m and 600 m; b) 300 m and 1000 m; c) 300 m and 2000 m; d) 600 m and 2000 m.
(TIF)
