# Supplementary figures and images for: GIS-based landform classification of Bronze Age archaeological sites on Crete Island
Source: PLoS One. 2017 Feb 21;12(2):e0170727. doi: 10.1371/journal.pone.0170727 (PMC5319673; doi:10.1371/journal.pone.0170727)

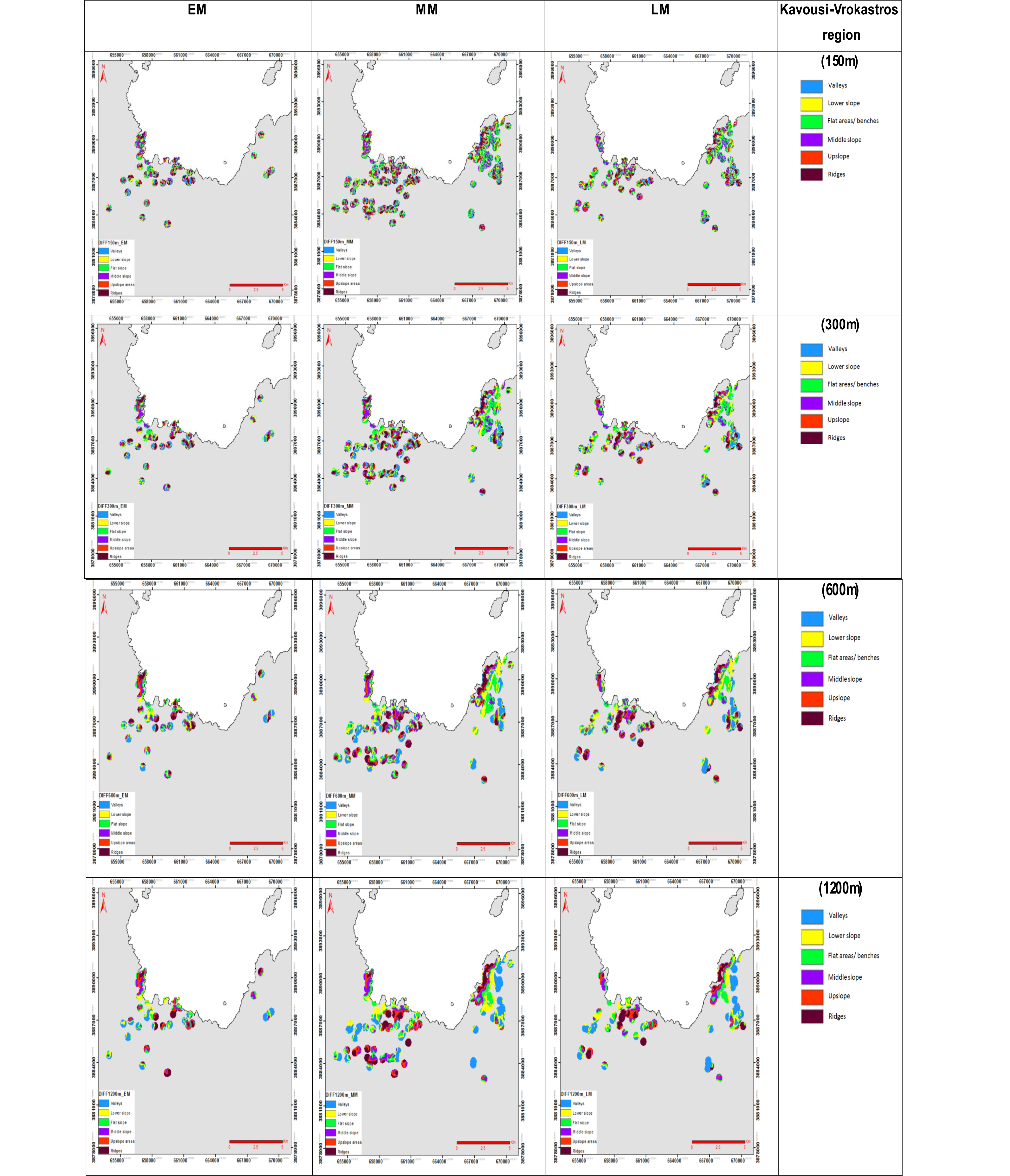

Supplement: S1 Fig — TPI or DIFF for EM, LM and MM period on Kavousi-Vrokastro region, with six morphologic classes for the neighbourhood sizes: a) 150 m; b) 300 m; c) 600 m; d) 1200 m. (TIF) [file pone.0170727.s001.tif]

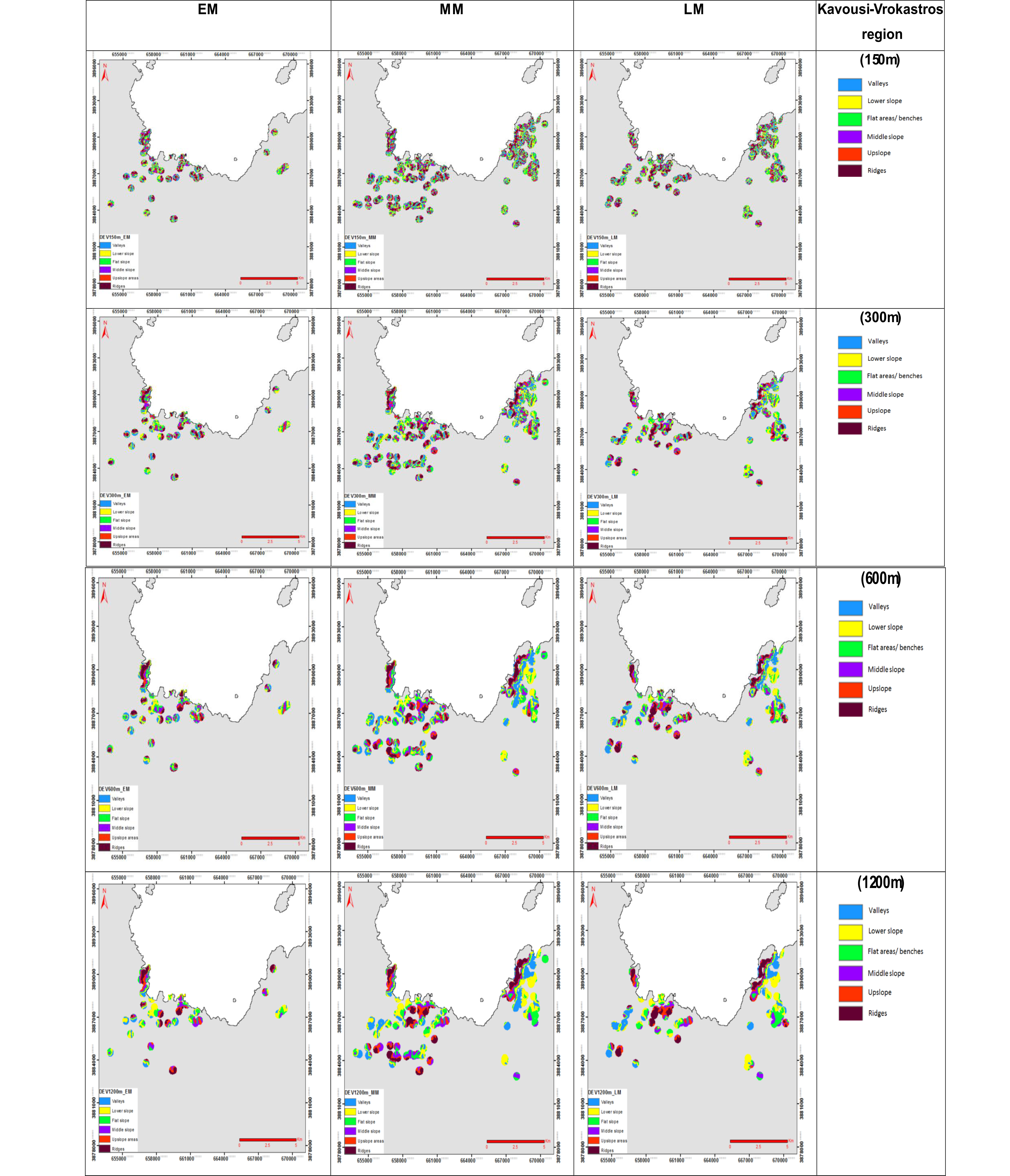

Supplement: S2 Fig — DEV for EM, LM and MM period on Kavousi-Vrokastro region, with six morphologic classes for the neighbourhood sizes: a) 150 m; b) 300 m; c) 600 m; d) 1200 m. (TIF) [file pone.0170727.s002.tif]

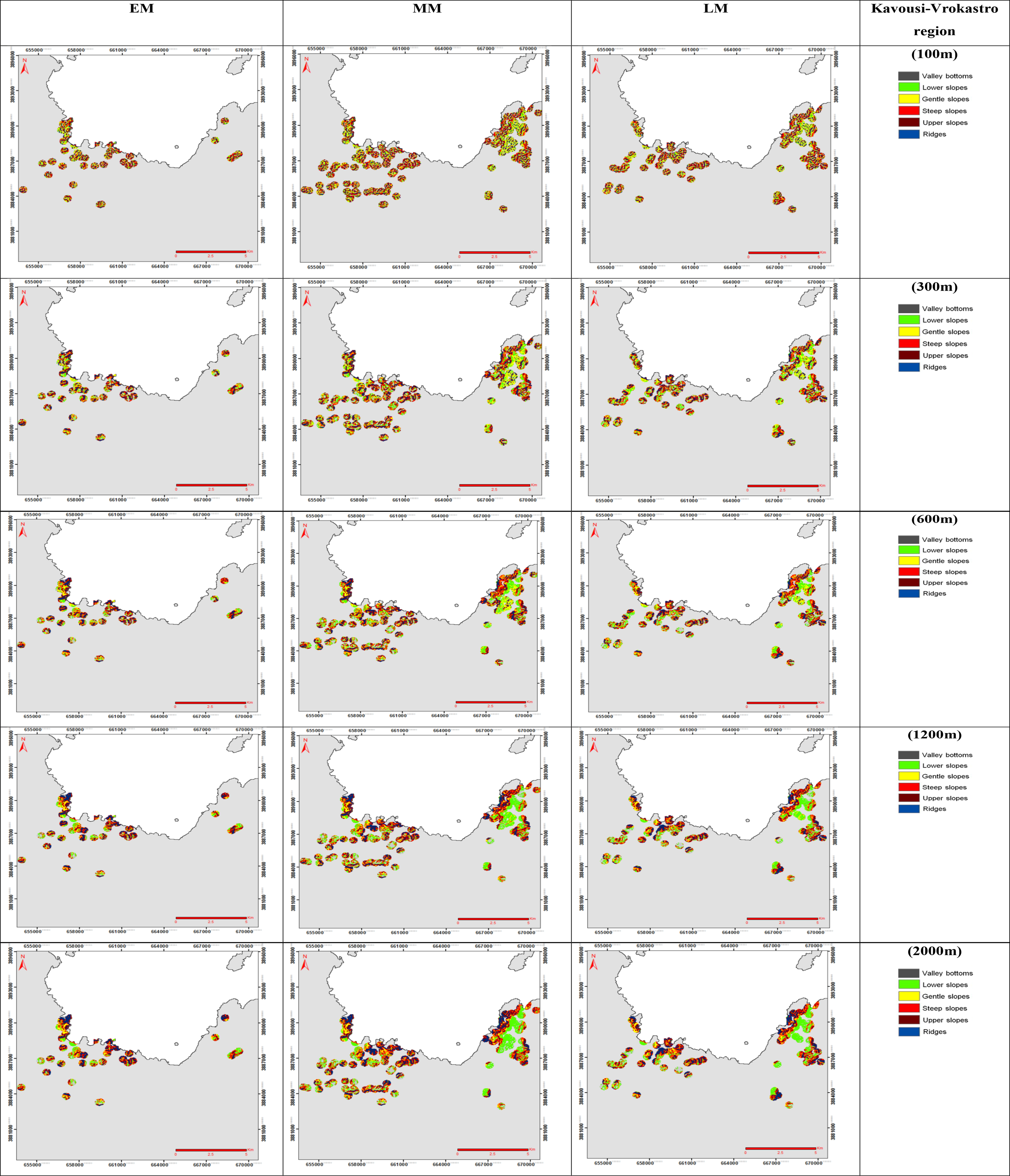

Supplement: S3 Fig — Slope position classification based on TPI of the case study sites of Kavousi-Vrokastro, for EM, LM and MM periods, with six morphological classes for the neighbourhood sizes: a) 100 m; b) 300 m; c) 600 m; d) 1200 m; e) 2000 m. (TIF) [file pone.0170727.s003.tif]

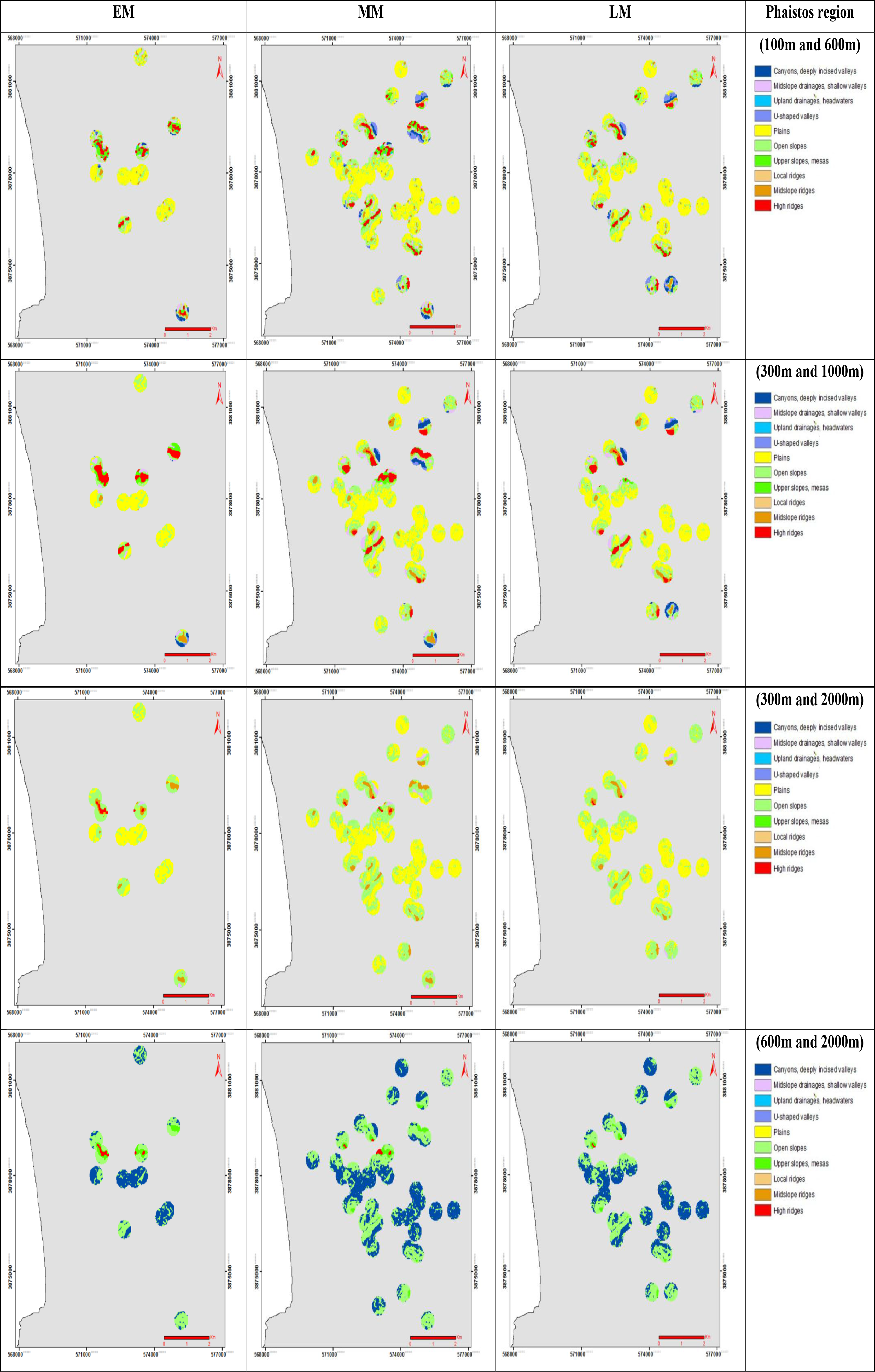

Supplement: S4 Fig — Landform classification based on TPI of the case study sites of Phaistos, for EM, LM and MM periods, with ten landform types for the combined neighbourhood sizes: a) 100 m and 600 m; b) 300 m and 1000 m; c) 300 m and 2000 m; d) 600 m and 2000 m. (TIF) [file pone.0170727.s004.tif]
